# Prevalence of Ischemic Heart Disease and Management of Coronary Risk in Daily Clinical Practice: Results from a Mediterranean Cohort of HIV-Infected Patients

**DOI:** 10.1155/2014/823058

**Published:** 2014-08-07

**Authors:** Patricia Echeverría, Pere Domingo, Josep-María Llibre, Mar Gutierrez, Gracia Mateo, Jordi Puig, Anna Bonjoch, Nuria Pérez-Alvarez, Guillem Sirera, Bonaventura Clotet, Eugenia Negredo

**Affiliations:** ^1^“Lluita contra la Sida” Foundation, Hospital Universitari Germans Trias i Pujol, Badalona, 08916 Barcelona, Spain; ^2^Universitat Autònoma de Barcelona, Barcelona, Spain; ^3^Hospital Universitari Santa Creu i Sant Pau, Barcelona, Spain; ^4^Statistics and Operations Research, Technical University of Catalunya, Barcelona, Spain; ^5^Universitat de Vic-Universitat Central de Catalunya, 08570 Barcelona, Spain; ^6^IrsiCaixa Foundation, Barcelona, Spain

## Abstract

*Background*. There are conflicting data on the prevalence of coronary events and the quality of the management of modifiable cardiovascular risk factors (CVRF) in HIV-infected patients. *Methods*. We performed a retrospective descriptive study to determine the prevalence of coronary events and to evaluate the management of CVRF in a Mediterranean cohort of 3760 HIV-1-infected patients from April 1983 through June 2011. *Results*. We identified 81 patients with a history of a coronary event (prevalence 2.15%); 83% of them suffered an acute myocardial infarction. At the time of the coronary event, CVRF were highly prevalent (60.5% hypertension, 48% dyslipidemia, and 16% diabetes mellitus). Other CVRF, such as smoking, hypertension, lack of exercise, and body mass index, were not routinely assessed. After the coronary event, a significant decrease in total cholesterol (*P* = 0.025) and LDL-cholesterol (*P* = 0.004) was observed. However, the percentage of patients who maintained LDL-cholesterol > 100 mg/dL remained stable (from 46% to 41%, *P* = 0.103). Patients using protease inhibitors associated with a favorable lipid profile increased over time (*P* = 0.028). *Conclusions*. The prevalence of coronary events in our cohort is low. CVRF prevalence is high and their management is far from optimal. More aggressive interventions should be implemented to diminish cardiovascular risk in HIV-infected patients.

## 1. Introduction

The increased life expectancy of the HIV-1-infected population means that physicians are increasingly being faced with previously unrecognized comorbid conditions and antiretroviral-related complications. Atherosclerosis and cardiovascular events, loss of renal function, osteopenia/osteoporosis, and non-AIDS-defining cancers are some of the emerging conditions observed in large observational cohorts, and their incidence seems to be higher than in the general population [1–7]. In addition, not only is HIV infection associated with AIDS-defining neurologic conditions with severe CD4 depletion, but also HIV-associated neurocognitive disorders seem more common in HIV-infected individuals despite achieving a successful immune recovery.

Several studies, including the data collection on adverse events of anti-HIV drugs (D.A.D.) study, have demonstrated an increased rate of premature cardiovascular events, including myocardial infarction, in HIV-1-infected individuals exposed to specific individual antiretroviral drugs [8–14]. Traditional cardiovascular risk factors (CVRF) have at least a similar impact on cardiovascular disease (CVD) in this population as they do in the general population [15–17]. However, both HIV replication and antiretroviral therapy may contribute independently to an increased risk of CVD [4, 8–14, 18–20]. Several hypotheses have been formulated to explain a potential increase in the incidence of premature aging and coronary events in these patients [3, 4, 18–23]. Some mechanisms are related to antiretroviral therapy, such as the mitochondrial dysfunction and oxidative stress induced by thymidine analogues [10–12] or protease inhibitor- (PI-) related dyslipemia [8, 9, 13, 14], while the virus itself contributes to increased cardiovascular risk by a chronic inflammatory effect or a direct effect on endothelial and other cells [4, 20, 21]. These factors, together with the increased incidence of traditional CVRF in HIV-1-infected patients, could pave the way to the development of coronary events [4, 8–14, 16, 19–24].

Encouraging data suggest that the incidence of ischemic heart disease in HIV-1-infected patients could be decreasing in recent years, probably due to the use of new antiretroviral regimens associated with higher suppression rates of HIV-1 replication and more favorable lipid profiles [25]. However, data on the excellence in the control of modifiable CVRF in HIV-infected individuals are scant.

We assessed the management of CVRF in the routine clinical practice in HIV-infected subjects already diagnosed for a coronary event.

## 2. Methods

### 2.1. Study Design and Population

We performed a retrospective descriptive study to identify all HIV-1-infected patients with a previous coronary event recorded in the database of the HIV unit, internal medicine and cardiology departments of two tertiary hospitals in Barcelona, Spain, from April 1983 through June 2011. The database contained information on 3,760 HIV-1-infected patients in routine follow-up. In addition, the causes of mortality in both cohorts during the period of study (from April 1983 to June 2011) have been checked to identify those related to a cardiovascular event.

A coronary event was defined as a definite or probable acute myocardial infarction or reinfarction, angina, percutaneous coronary angioplasty/stenting, coronary bypass surgery, target vessel revascularization for restenosis, stent thrombosis, and death from ischemic coronary disease according to the criteria of the World Health Organization Multinational Monitoring of Trends and Determinants in Cardiovascular Disease Project (MONICA) [26].

Cases of myocardial infarction were categorized as fatal or nonfatal.

### 2.2. Study Objectives and Endpoints

We determined the prevalence of coronary events by assessing the numbers of subjects per 1000 HIV-infected patients in a Mediterranean cohort to identify all HIV-1-infected patients with a previous coronary event recorded in the database from April 1983 until June 2011.

To evaluate changes in modifiable CVRF before and after the coronary event, we compared the prevalence of the modifiable CVRF in the clinic at three time points: before initiating antiretroviral therapy, at the time of the coronary event, and after the event (the last available visit until June 2011).

Finally, we compared the Framingham risk score at the time of the coronary event and at last follow-up visit.

### 2.3. Assessments

We recorded the following information: sociodemographic features (age, gender, and race); HIV-related data (time since HIV diagnosis, risk behavior, previous AIDS diagnosis defined according to the Centers for Disease Control and Prevention category C [27], time on antiretroviral therapy, time on HAART, nadir CD4 T-cell count, and cumulative exposure to antiretroviral drugs such as nonnucleoside reverse transcriptase inhibitors [NNRTI], the nucleoside reverse transcriptase inhibitor [NRTI] abacavir, and protease inhibitors (PIs)); personal and family history of hypertension, diabetes mellitus, dyslipidemia, previous cardiovascular events, nephropathy, hepatitis coinfection, use of concomitant therapy (treatment for dyslipidemia, hypertension, and diabetes mellitus); body mass index; the practice of regular exercise (at least 3 hours per week of cardiovascular exercise); consumption of drugs, coffee, alcohol, and tobacco.

In addition, at each of the three time points mentioned above, we recorded laboratory data performed after at least 8 hours of fasting (plasma HIV-1 RNA levels, lymphocyte CD4 T-cell count, total cholesterol, low-density lipoprotein [LDL] cholesterol, high-density lipoprotein [HDL] cholesterol, triglycerides, glomerular filtration rate, and glucose level) and the Framingham risk score. Glycosylated hemoglobin was not considered because it was not a routine laboratory parameter.

Cardiovascular risk was classified according to the Framingham score as low (<10%), moderate (10%–20%), or high (≥20%).

Hypertension was defined as systolic blood pressure ≥140 mm Hg, diastolic blood pressure ≥90 mm Hg, or the need for antihypertensive drugs. Patients were considered to have dyslipidemia if they were taking lipid-lowering drugs or when the total cholesterol level was >200 mg/dL, LDL-cholesterol level was >100 mg/dL (criteria for HIV-infected patients according to the “European Guidelines on cardiovascular disease prevention in clinical practice” [28]), and/or triglycerides were >200 mg/dL.

Antiretroviral drugs were classified according to their impact on lipid metabolism in two groups: those with a negative impact on triglyceride and cholesterol levels (lopinavir, indinavir, and fosamprenavir) and those with a neutral effect (atazanavir, saquinavir, darunavir, and NNRTIs).

### 2.4. Statistical Analysis

Demographic and clinical parameters were expressed as mean (SD), median (interquartile range, IQR), or frequency and percentage, as appropriate.

Continuous repeated measurements were compared using the *t*-test, Wilcoxon test, or Friedman test; proportions were compared using the McNemar test or Cochran test.

Univariate *P* values <0.05 were considered significant. All statistical analyses were performed using SPSS software, version 15.0.0 (SPSS Inc, Chicago, Illinois, USA).

## 3. Results

We identified 81 HIV-1-infected patients with a previous coronary event in a cohort of 3760 patients. The prevalence of a coronary event was 2.15%.

### 3.1. Coronary Event

The most frequent event was acute myocardial infarction (67 patients, 82.7%), followed by unstable angina (10 patients, 12%), reinfarction, or angor following acute myocardial infarction (4 patients, 5.3%).

Coronary angiography was undertaken in 43 patients (53%) and revealed stenosis with single vessel involvement in 19 of them (41.9%) and 2 or more vessels in 25 (58.1%).

Forty-six patients (57%) received invasive treatment: 24 of them (30%) received a percutaneous angioplasty, 18 (22%) open bypass surgery, and 4 (5%) vessel revascularization for restenosis and stent thrombosis. The remaining 35 patients (43%) only required medical treatment.

The mortality rate registered in our cohort among subjects with previous coronary events at the end of study (June 2011) was 13.6% (11/81). Of these, 9 (11%) patients died due to noncardiovascular causes such as kidney failure, liver failure, opportunistic infections, brain subdural hematomas, respiratory infections, and others, and only two patients (2.5%) died as a result of a coronary event (one of them was related to illicit drug use, cocaine).

### 3.2. Patient Data at the Time of the Coronary Event

Table 1 shows the traditional CVRF and the epidemiologic and HIV-related characteristics of the patients at the time of the coronary event.

From 81 HIV-1-infected patients with a previous coronary event, overall 86% of them were males, the median age was 48 (40, 57) years, and 38% of patients were >50 years old.

As for CVRF, 60.5% of patients had a history of hypertension, 48% dyslipidaemia, and 16% diabetes mellitus; of them, 12.2%, 23.1%, and 11.4% required treatment for these conditions, respectively, but they did not receive it or this information was not available in the patient's records. Forty-four percent of patients were current smokers and 9.8% had a body mass index >25. The information in the medical records revealed that only 3 patients (3.7%) were questioned about physical exercise.

At the time of the coronary event, the median follow-up since diagnosis of HIV infection was 14.9 (10.4; 19.2) years, the median time (IQR) from starting antiretroviral treatment until the coronary event and the median time from the coronary event until the last follow-up visit (June 2011) were 8 (5; 12) and 7 (4; 11) years, respectively, and the median time of receiving antiretroviral treatment was 12.7 (9.3, 16.2) years. Regarding antiretroviral treatment, 33 patients (41%) were receiving a PI-based regimen: 22 patients (67%) were receiving PI-based regimen with an unfavorable lipid profile and 11 (33%) a PI-based regimen with favorable lipid profile; 26 (32%) were receiving a NNRTI-based regimen: 11 patients were on efavirenz (42%) and 15 on nevirapine (58%); 6 patients (7%) were receiving other regimens (integrase inhibitors, CCR5 antagonist) and 17 patients (20%) were not taking antiretroviral treatment.

At the time of the coronary event, the calculation of Framingham score was only possible in 12 patients (15%) due to the lack of some necessary parameters (mainly determination of HDL-cholesterol levels, tobacco, and blood pressure). Among those 12 patients, the CVR was classified as high in 1 patient (8%), moderate in 8 (67%) patients, and low in 3 (25%) patients.

### 3.3. Changes in Modifiable Cardiovascular Risk Factors at the Three Time Points

Changes among the three time points of the study in smoking habit, exercise, body mass index, use of lipid-lowering drugs, and antihypertensive and oral antidiabetic drugs/insulin were not routinely assessed or correctly registered in the routine follow-up (were not coded). This information was mainly available in the clinical record at the time of the coronary event, but not in other points in many patients. Similarly, in most cases, it was not possible to evaluate variations in Framingham score, due to the lack of necessary information to calculate it.

Metabolic parameters at the three time points are summarized in Table 2.

Levels of LDL-cholesterol and HDL-cholesterol were only determined in 18.5% of subjects at the baseline visit (previous antiretroviral therapy); 46% of them had an LDL-cholesterol level of >100 mg/dL. At the time of the coronary event, an LDL-cholesterol determination was available for 47% of patients, and it was >100 mg/dL in 45% of them. Finally, at the last available visit (after the coronary event), LDL-cholesterol determination was available for 73% of patients, and in 41% it was >100 mg/dL.

A significant decrease from the time of the coronary event to the last visit available was observed in total cholesterol [from 182 ((152, 230) mg/dL to 174 (147, 205), *P* = 0.025] and LDL-cholesterol [from 104 (98, 194) mg/dL to 95 (86, 124), *P* = 0.004].

No significant differences among the three time points were observed in HDL-cholesterol, total/HDL-cholesterol ratio, triglyceride levels, and glycemia.

The percentage of patients receiving PIs with an unfavorable lipid profile decreased from 67% during the coronary event to 37% at the last follow-up visit (*P* = 0.028), while change to PIs with better lipid profile was from 33% to 63% (*P* = 0.028) (Table 3).

The rate of patients with suppressed HIV-1 RNA viral load significantly increased after the coronary event (*P* < 0.000).

## 4. Discussion 

The prevalence of coronary events in our cohort of HIV-1-infected subjects (2.15%) was lower than that observed by other groups [29, 30]. The most frequent coronary events were acute myocardial infarction and unstable angina, and 2.5% of them were fatal.

Traditional CVRF were highly prevalent in patients with a history of coronary heart disease at the time of the event. The percentage of patients routinely undertaking fasting lipid profiles significantly increased after the coronary event and a decrease in total and LDL-cholesterol levels was observed after the event. Nevertheless, many patients (41%) did not achieve the recommended target in lipid parameters. Additionally, other CVRF such as smoking, lack of exercise, or hypertension were not regularly controlled.

HIV-1-infected individuals receiving HAART have a higher risk for ischemic heart disease than the general population [1, 8–14, 17, 19, 24, 31, 32]. Previous studies report incidence rates ranging from 1.53 to 6.01 cases per 1000 person-years in patients exposed to protease inhibitors [9] and a progressive increase associated with the prolonged exposure to antiretroviral therapy, particularly in patients on a PI-containing regimen [8, 9, 12–14].

A meta-analysis including 19 studies evaluating cardiovascular and cerebrovascular events in Spanish population showed that the prevalence of angina in Spain was about 7%, while it was 2.6% in men and 3.5% in women aged from 25 to 74 years in another cohort [33–36]. In our HIV-1-infected population, on the other hand, the prevalence of coronary events was slightly lower (2.15%). Although the search was exhaustive, it could be the possibility that not all cases were recorded due to the retrospective design of the study.

The pathogenic mechanism of premature atherosclerosis in HIV-1-infected individuals has not been clearly defined, although it is considered a multifactorial process. HIV infection and antiretroviral therapy might accelerate atherogenesis, as do traditional risk factors. First, a chronic systemic inflammatory state, endothelial dysfunction, and prothrombotic state caused by the virus itself contribute to the pathogenesis of coronary disease [4, 18–22]; therefore, viral suppression should be maintained to reduce vascular damage [20, 37, 38]. The higher prevalence of coronary atherosclerosis in young asymptomatic men with long-standing HIV infection in comparison with non-HIV-1-infected subjects supports the role of the virus [4, 18, 19, 32]. In our group of patients, viral suppression was present in only half of them, at time of coronary event. Second, although seemingly paradoxical, antiretroviral therapy could also increase the risk of CVD [1, 8–14, 20], partly explained by PI-related metabolic and lipid changes [9, 39, 40]. Both HIV infection and antiretroviral therapy may contribute independently to the increase of the cardiovascular risk and acceleration of the pathogenesis of other conditions not related to AIDS. Thus, physicians must be more proactive with the management of HIV and interventions related to switching treatments to drugs with better profile to limit the potential cardiovascular damage.

Finally, traditional CVRF and smoking, cocaine use, or coinfection with hepatitis C virus (all of which are proatherogenic factors) are more common in HIV-1-infected individuals than in the general population, [15–17, 34, 41]. The very high rates of smoking, hypertension, dyslipidemia, and diabetes we observed in our group were consistent with those of other cohorts of HIV-1-infected patients with myocardial infarction [19, 31, 41, 42].

Fortunately, the incidence of cardiovascular events in the HIV-1-infected population has fallen in recent years. This is possibly as a result of more aggressive management of the risk of CVD, the use of drugs with better cardiovascular profile [25], and the viral suppression in almost all HIV-1-infected patients in our setting that has limited the damage of sustained viral replication in the vascular system. A higher proportion of our patients showed viral suppression in the last visit than at time of the coronary event. In addition, the percentage of patients in our cohort receiving antiretroviral drugs with a negative effect on lipid metabolism has decreased over time.

Surprisingly, in our cohort, the number of patients who were receiving treatment for concomitant conditions (lipid-lowering agents, antihypertensive drugs, or antidiabetic drugs) was extremely high. Similarly, LDL- and HDL-cholesterols were more frequently determined during recent years, indicating increased efforts in achieving a better control of lipid metabolism. However, treatment for dyslipidemia was suboptimal or poorly controlled in most cases. Lipid levels improved after the coronary event, although almost half (41%) of the patients maintained an LDL-cholesterol level > 100 mg/dL after the coronary event. Second, other CVRF, such as smoking, hypertension, lack of exercise, and body mass index, are not routinely assessed. These data were not available in the clinical histories of most of our patients, suggesting poor monitoring of these risk factors in our clinical practice.

The mortality rate registered in our cohort among subjects with previous coronary events was 2.5%, slightly lower than that observed by other groups (4.2%) [30]. Although our search was exhaustive, we cannot rule out (given the retrospective study design) the possibility that some cardiovascular-related deaths were not included in our database or were attributed to “unknown causes” and have not been captured in the analysis.

The retrospective design of our study did not enable us to determine the real rate of some risk factors and thus evaluate changes. However, the study provides us a clear picture of real clinical practice. In addition, despite performing an exhaustive search, we cannot exclude the possibility of missed coronary events attended at other centers and not reported in our database. However, this rate would be low and is unlikely to drive a significant change in the study results.

In summary, the management of CVRF in our cohort was far from optimal, in subjects already diagnosed for prior CV major events. However, as this group is aging, we expect a progressive increase in the incidence of CVD paralleling the age group. Cardiovascular health plans are a priority if this increase is to be curbed and need to carefully monitor the CVRF to assess the effectiveness of such plans. Optimal selection of antiretroviral treatment, together with aggressive management of other modifiable risk factors, will diminish cardiovascular risk in HIV-1-infected patients.

## Figures and Tables

**Table 1:** Patient characteristics at the time of the coronary event.

Characteristics	HIV-infected patients (*n* = 81)
Age, years (median [IQR])	48 (40, 57)
Patients > 50 years (%)	38.3
Gender (male) (%)	86.1
Body mass index >25 (%)	9.8
Men who have sex with men (%)	38.6
HCV coinfection (%)	4.9
HBV coinfection (%)	12.3

HIV-related characteristics	
Time since diagnosis of HIV-infection, years (median [IQR])	14.9 (10.4, 19.2)
Cumulative exposure to antiretrovirals (median [IQR])	12.7 (9.3, 16.2)
Cumulative exposure to PIs (median [IQR])	5 (0.7, 8)
Cumulative exposure to NNRTIs (median [IQR])	3 (0.1, 7)
Cumulative exposure to abacavir (median [IQR])	2 (0, 11)
CD4 cells count/*µ*L (median [IQR])	224 (89, 422)
HIV-RNA ≤ 400 copies/mL (median [IQR])	46 (56.8%)
Lipodystrophy (including lipoatrophy) (%)	36.1

Cardiovascular risk factors	
Family history of coronary heart disease (%)	17 (20.5%)
Smoking (%)	36 (44%)
Cumulative exposure to tobacco smoke, years (mean [SD])	23 (9.4)
Hypertension (%)	49 (60.5)
∗Use of antihypertensive treatment (%)	43 (87.8)
Diabetes mellitus (%)	13 (16)
∗Use of antidiabetic treatment (%)	10 (76.9)
Dyslipidemia (%)	44 (48.1)
Serum total cholesterol, mg/dL (median [IQR])	182 (152, 230)
Serum HDL-cholesterol, mg/dL (median [IQR])	42 (35, 52)
Serum LDL-cholesterol, mg/dL (median, [IQR])	104 (98, 194)
Serum triglycerides, mg/dL (median, [IQR])	168 (106, 248)
∗Use of lipid-lowering agents (%)	39 (88.6)

Data was reported as median and interquartile range [IQR].

∗Percentages of patients using antihypertensive drugs, antidiabetic treatment or lipid-lowering agents were calculated considering the number of patients receiving that treatment with respect to those who required treatment (considering target parameters according to the guidelines).

**Table 2:** Changes in modifiable cardiovascular risk factors and Framingham score at the three time points of the study in the 81 included patients.

Laboratory data	Before starting ARV	At the time of the coronary event^a^	Last observation^b^	^ c^ *P* value (baseline to coronary event)	^ d^ *P* value (event to present)
CD4, cell/*µ*L (median [IQR])	224 (89, 422)	497 (384, 776)	559 (375, 767)	**0.000**	0.201
HIV-RNA, log (median [IQR])	4.6 (3.6, 5.3)	1.8 (1.4, 2.7)	1.4 (1.3, 1.7)	**0.000**	**0.000**
Serum total cholesterol, mg/dL (median [IQR])	185 (154, 213)	182 (152, 230)	174 (147, 205)	0.402	**0.025**
Serum HDL-cholesterol, mg/dL (median [IQR])	41 (30, 43)	42 (35, 52)	45 (33, 53)	0.155	0.974
Serum LDL-cholesterol, mg/dL (median [IQR])	102 (97, 189)	104 (98, 194)	95 (86, 124)	0.305	**0.004**
CT/HDL-cholesterol ratio mmol/L	4.3 (3.8, 5.6)	4.1 (3.1, 5.8)	4 (3.1, 5.2)	0.110	0.339
Serum triglycerides, mg/dL (median [IQR])	151 (106, 204)	168 (106, 248)	142 (95, 204)	0.778	0.111
Glycemia, mg/dL (median [IQR])	90 (85, 103)	97 (88, 108)	95 (88, 108)	0.140	0.299
Glomerular filtration rate, mL/min (median [IQR])	60 (60, 60)	60 (60, 60)	60 (60, 60)	0.317	0.317
Serum total cholesterol >200 mg/dL (*n*, %)	18 (49)	29 (45)	25 (35.2)	0.265	0.738
Serum HDL-cholesterol <40 mg/dL (*n*, %)	73 (90)	21 (44)	25 (39.7)	**0.001**	1.000
Serum LDL-cholesterol >100 mg/dL (*n*, %)	7 (46)	17 (45)	25 (41%)	—	0.103
Serum triglycerides >200 mg/dL (*n*, %)	15 (41)	30 (37)	26 (32.1)	**0.022**	**0.238**
Serum triglycerides >500 mg/dL (*n*, %)	1 (2.7)	4 (6.3)	3 (4.1)	0.118	0.193
Glycemia >110 mg/dL (*n*, %)	5 (15)	15 (23)	17 (24.3)	**0.001**	**0.52**
Framingham <10% (low) (*n*, %)	2 (50)	3 (25)	11 (44)	—	—
Framingham 10%–20% (moderate) (*n*, %)	2 (50)	8 (67)	8 (32)	—	—
Framingham >20% (high) (*n*, %)	—	1 (8)	6 (24)	—	—

^a^Median time (IQR) from starting antiretroviral treatment until the coronary event: 8 (5; 12) years; ^b^median time (IQR) from the coronary event until the last follow-up visit (June 2011): 7 (4; 11) years.

^c^ *P* value denotes change from baseline to the time of the coronary event; ^d^ *P* value denotes change from the coronary event to the last follow-up visit.

CT: total cholesterol; HDL: high-density lipoprotein; LDL: low-density lipoprotein.

**Table 3:** Current antiretroviral therapy during coronary event and at the last follow-up visit.

	Coronary event *n* (%)	Last follow-up visit^a^ *n* (%)	*P* value^b^
Without treatment (*n* = 28)	17 (20)	11 (14)	0.3
**PI-based regimen** (*n* = 68)	33 (41)	35 (43)	0.87
PI (with an unfavorable lipid profile)	22 (67)	13 (37)	**0.028**
Indinavir/r	7 (21)	2 (6)	0.13
Lopinavir/r	15 (45)	10 (29)	0.23
Fosamprenavir/r	0	1 (3)	1
PI (with a favorable lipid profile)	11 (33)	22 (63)	**0.028**
Saquinavir/r	2 (6)	0	0.45
Atazanavir/r	6 (18)	11 (31)	0.33
Darunavir/r	3 (9)	11 (31)	**0.048**
**NNRTI-based regimen** (*n* = 57)	26 (32)	31 (38)	0.51
Nevirapine	15 (58)	14 (45)	0.5
Efavirenz	11 (42)	17 (55)	0.5
Other treatments (*n* = 10)	6 (7)	4 (5)	0.74
Without treatment	0	5 (33)	1

Nucleoside reverse transcriptase inhibitor			
Abacavir	15 (52)	14 (48)	1

^a^Median time (IQR) from the coronary event until the last follow-up visit (June 2011): 7 (4; 11) years.

^b^ *P* values express intragroup differences between coronary event time and last visit (after event).

PI: protease inhibitors; NNRTI: nonnucleoside analog reverse-transcriptase inhibitors.
